# Supplementary material for: The mediation of perceived risk’s impact on destination image and travel intention: An empirical study of Chengdu, China during COVID-19
Source: PLoS One. 2022 Jan 7;17(1):e0261851. doi: 10.1371/journal.pone.0261851 (PMC8741017; doi:10.1371/journal.pone.0261851)
Supplement: S1 Table — (DOCX) [file pone.0261851.s001.docx]

**S1 Table. Similar studies in China.**

| Researcher(s) | Study site | Findings and relevant conclusions |
| --- | --- | --- |
| Zhu (2020)[1] | China | Potential participants paid most attention to the performance realization and time cost of scenic spots, while the psycho-social risk posed by COVID-19 had little impact. With regard to travel intention and recommendation intention, the negative impacts of risk aversion attitude were more considerable compared to risk perception. Meanwhile, the parallel mediating effect of risk perception and risk aversion attitude in rural tourism needed to be taken into consideration together. |
| Ahmed (2020)[2] | China | Higher rate of anxiety, depression, hazardous and harmful alcohol use, and lower mental wellbeing than usual ratio. |
| Luo (2020)[3] | Hong Kong | The fear of COVID-19, travel anxiety and risk attitude negatively impact travel intention. Furthermore, travel anxiety and risk attitude moderate the indirect impacts between fear of COVID-19 and travel intention. |
| Wang (2020)[4] | Jilin City (Jilin Province) | SARIMA model is established to analyze the impact of COVID-19 on China’s tourism revenue, and it is concluded that China should focus on improving the quality and upgrading of domestic tourism in the future. The Challenges and Countermeasures for Tourism Industry of China were discussed. |
| Wang (2020)[5] | Henan Province | The impact and formation mechanism of the Novel Coronavirus Pneumonia (NCP) on the tourism industry in Henan Province were investigated. In the period of “post-NCP “,tourism development will return to the nature of health, people travel slowly and hot, tourism recovery will be a difficult recovery process. |
| Yang (2020)[6] | Hubei Province | During the different stages of COVID-19, including discovery, outbreak, and subsidence, the overall impact of urbanization on the economy in Hubei Province was first positive, then became negative, and finally gradually increased. This process had significant industrial and urban heterogeneity, which was mainly manifested in losses in tourism and catering industries that were significantly greater than those in the audio-visual entertainment and digital office industries. |
| Qiao (2021)[7] | China | Mass media had a positive influence on South Korean residents’ perception of China’s image, a negative influence on residents’ concerns, and a positive influence on residents’ behavioral intentions for travel overseas. |
| Jose (2021)[8] | Macao | A survey instrument was designed to evaluate resident satisfaction with six enforcement actions taken by the Macao government during the COVID-19 pandemic. Each factor had a different effect on satisfaction and trust that influenced the resident support for tourism recovery. |
| Lu (2021)[9] | Sichuan Province | The hospitality industry sector, the most severely affected sector in five industries, by cash flow pressure. |
| Sun (2021)[10] | Hainan Province, Hubei Province, Shanghai, Beijing | Four game models for epidemic prevention and control and tourism recovery are proposed, combined with the Logistic growth curve, to predict the recovery of passenger traffic in different cases, and use it as tourism popularity to deduce tourism recovery. The recovery of passenger traffic is subject to the epidemic prevention and control policy, but the policy. The intensity of the impact on passenger traffic is less than that of the epidemic. |
| Yang (2021)[11] | Hengyang (Hunan Province) | Travel time perception, tourism function perception, tourism satisfaction, tourism risk perception and tourism willingness will affect tourism well-being. |

# References

Zhu H, Deng F. How to Influence Rural Tourism Intention by Risk Knowledge during COVID-19 Containment in China: Mediating Role of Risk Perception and Attitude. Int J Environ Res Public Health. 2020 May 18;17(10):3514. doi: 10.3390/ijerph17103514. PMID: 32443430.

Ahmed MZ, Ahmed O, Aibao Z, Hanbin S, Siyu L, Ahmad A. Epidemic of COVID-19 in China and associated Psychological Problems. Asian J Psychiatr. 2020 Jun;51:102092. doi: 10.1016/j.ajp.2020.102092. Epub 2020 Apr 14. PMID: 32315963.

Luo JM, Lam CF. Travel Anxiety, Risk Attitude and Travel Intentions towards "Travel Bubble" Destinations in Hong Kong: Effect of the Fear of COVID-19. Int J Environ Res Public Health. 2020 Oct 27;17(21):7859. doi: 10.3390/ijerph17217859. PMID: 33120949.

Wang Q S, He Z X.On the Challenges and Countermeasures for Tourism Industry of China in the Post Epidemic Period of COVID －19: A Case Study of Jilin City，Jilin Province.China Soft Science.2020;(S1):147-154.doi:CNKI:SUN:ZGRK.0.2020-S1-018.

Wang S H, Wang lu, Wang mengyin, Wang wei.Study on Impact Characterization and Influence Mechanism of Novel Coronavirus Pneumonia on Tourism Industry in Henan Province.Areal Research and Development.2020;39(2):1-7.doi:CNKI:SUN:DYYY.0.2020-02-001.

Yang W, Wang X, Zhang K, Ke Z. COVID-19, Urbanization Pattern and Economic Recovery: An Analysis of Hubei, China. Int J Environ Res Public Health. 2020 Dec 21;17(24):9577. doi: 10.3390/ijerph17249577. PMID: 33371455.

Qiao G, Zhao XL, Xin L, Kim S. Concerns or Desires Post-Pandemic: An Extended MGB Model for Understanding South Korean Residents' Perceptions and Intentions to Travel to China. Int J Environ Res Public Health. 2021 Mar 4;18(5):2542. doi: 10.3390/ijerph18052542. PMID: 33806384.

Jose Weng Chou Wong, Ivan Ka Wai Lai. Effect of government enforcement actions on resident support for tourism recovery during the COVID-19 crisis in Macao, China. Asia Pacific Journal of Tourism Research. 2021 Jun 30;26(9): 973-87.doi:10.1080/10941665.2021.1940224.

Lu Li,Peng Junlin,Wu Jing,Lu Yi. Perceived impact of the Covid-19 crisis on SMEs in different industry sectors: Evidence from Sichuan, China. International Journal of Disaster Risk Reduction.2021Mar;55:102085. doi:10.1016/J.IJDRR.2021.102085.

Sun G N,Gan X R. Recovery and forecast of tourism in typical provinces of China in the post-epidemic era: taking the monthly traffic volume data of Hainan, Hubei, Shanghai and Beijing as an example. Journal of Shaanxi Normal University (Natural Science Edition). 2021; 49(6)： 9-20. doi： 10. 15983/j. cnki. jsnu. 2021. 04. 014.

Yang J H, Zhang J H, Chu G.The logic of quality perception and well-being enhancement in tourist destinations under covid-19: based on the survey of tourism activities in the surrounding areas of hengyang residents and intersubjective introspection.Human geography.2021;36(3):167-174. doi:10.13959/j.issn.1003-2398.2021.03.018.
