# Supplementary material for: The mediation of perceived risk’s impact on destination image and travel intention: An empirical study of Chengdu, China during COVID-19
Source: PLoS One. 2022 Jan 7;17(1):e0261851. doi: 10.1371/journal.pone.0261851 (PMC8741017; doi:10.1371/journal.pone.0261851)
Supplement: S1 File — (DOCX) [file pone.0261851.s002.docx]

**1. Your gender： [Choose one]**

| ○Male | ○Female |  |  |  |  |  |  |
| --- | --- | --- | --- | --- | --- | --- | --- |

**2. Your age： [Choose one]**

| ○Under 18 | ○18 – 25 | ○26 – 30 | ○31 – 40 | ○41 – 50 | ○51 – 60 | ○≥61 |
| --- | --- | --- | --- | --- | --- | --- |

**3. Your marital status [Choose one]**

| ○Single |
| --- |
| ○Married |

**4. Your educational level [Choose one]**

| ○Middle school/below |
| --- |
| ○High school/equivalent |
| ○Bachelor’s/professional degree |
| ○Master’s/doctorate degree |

**5. Which city are you from? [Fill in]**

_________________________________

**6. Occupation: [Choose one]**

| ○Full-time student  ○Manufacturing personnel  ○Sales personnel  ○Marketing/Public Relations personnel  ○Customer Service personnel  ○Administrative/Logistics personnel  ○Human Resources  ○Finance/auditing personnel  ○Civilian/Clerk  ○Technical/R&D personnel  ○Management personnel  ○Teacher  ○Consultant  ○Professional (e.g. accountant, lawyer, architect, medical professional, journalist)  ○Other |
| --- |

**7. Industry： [Choose one]**

| ○IT/Software and Hardware Services/e-commerce/Internet Operations |
| --- |
| ○Fast-moving Consumer Goods (food/beverage/cosmetics) |
| ○Wholesale/Retail |
| ○Clothing/Textiles/Leather |
| ○Furniture/Crafts/Toys |
| ○Education/Training/Research/Academies |
| ○Home Appliances |
| ○Communications/Telecom Operations/Network Equipment/Value-added Services |
| ○Manufacturing |
| ○Automobiles and Automotive Parts |
| ○Dining/Entertainment/Travel/Hospitality/Life Services |
| ○Office Supplies and Equipment |
| ○Accounting/Auditing |
| ○Legal |
| ○Banking/Insurance/Securities/Investment Banking/Venture Funds |
| ○Electronic Technology/Semiconductors/Integrated Circuits |
| ○Instrumentation/Industrial Automation |
| ○Trade/Imports and Exports |
| ○Machinery/Equipment/Heavy Industry |
| ○Pharmaceuticals/Bioengineering/Medical Equipment/Apparatus |
| ○Medical/Nursing/Health/Hygiene |
| ○Advertising/PR/Media/Arts |
| ○Publishing/Printing/Packaging |
| ○Real Estate Development/Construction Engineering/Decoration/Design |
| ○Property Management/Commercial Center |
| ○Intermediary/Consulting/Headhunting/Certification |
| ○Transportation/Shipping/Logistics |
| ○Aerospace/Aviation/Energy/Chemicals |
| ○Agriculture/Fishing/Forestry |
| ○Other Industries |

8. **Which describes your past travel experience? [Choose one]**

| ○Have never traveled |
| --- |
| ○Have traveled once |
| ○Have traveled multiple times |

**9. Have you have participated in tourism activities since the outbreak of COVID-19? [Choose one]**

| ○Yes |
| --- |
| ○No |

**10. How likely do you feel the following risks are to exist or occur? [Choose one for each row]**

|  | Very unlikely | Unlikely | Neutral | Likely | Very likely |
| --- | --- | --- | --- | --- | --- |
| Human-made crises or natural disasters (earthquakes, mudslides, etc.) will occur at tourism sites | ○ | ○ | ○ | ○ | ○ |
| Public security incidents will occur at tourism sites | ○ | ○ | ○ | ○ | ○ |
| I will get sick during travel, e.g. with COVID-19 | ○ | ○ | ○ | ○ | ○ |
| The destination has poor infrastructure | ○ | ○ | ○ | ○ | ○ |
| The destination has poor sanitation | ○ | ○ | ○ | ○ | ○ |
| Traffic is inconvenient at the tourism destination | ○ | ○ | ○ | ○ | ○ |
| During the trip, actual costs will exceed expectations | ○ | ○ | ○ | ○ | ○ |
| Quarantine measures put in place for COVID-19 will entail time-related costs | ○ | ○ | ○ | ○ | ○ |
| Travel restrictions put in place for COVID-19 will mean that certain experiences are off-limits | ○ | ○ | ○ | ○ | ○ |
| If I travel to Chengdu during this period, others may think negatively of me | ○ | ○ | ○ | ○ | ○ |
| If I travel to Chengdu during this period, others will criticize me | ○ | ○ | ○ | ○ | ○ |
| If I travel to Chengdu during this period, friends and family members will not support my trip | ○ | ○ | ○ | ○ | ○ |
| Tourism activities will be unable to meet my requirements for relaxation | ○ | ○ | ○ | ○ | ○ |
| The quality of tourism services will not meet expectations | ○ | ○ | ○ | ○ | ○ |
| There are not as many tourism products as expected | ○ | ○ | ○ | ○ | ○ |
| I will feel worried traveling during the COVID-19 period | ○ | ○ | ○ | ○ | ○ |
| I will feel anxiety traveling during the COVID-19 period | ○ | ○ | ○ | ○ | ○ |
| I will feel nervous traveling during the COVID-19 period | ○ | ○ | ○ | ○ | ○ |

**11. What is your attitude towards risk aversion? [Choose one for each row]**

|  | Strongly disagree | Disagree | Neutral | Agree | Strongly agree |
| --- | --- | --- | --- | --- | --- |
| Before traveling, I will gather more information about the destination | ○ | ○ | ○ | ○ | ○ |
| I will buy travel insurance | ○ | ○ | ○ | ○ | ○ |
| I will get a vaccine | ○ | ○ | ○ | ○ | ○ |

**12. How does media influence your choice of travel destination? [Choose one for each row]**

|  | Strongly disagree | Disagree | Neutral | Agree | Strongly agree |
| --- | --- | --- | --- | --- | --- |
| I consider media opinions when selecting my vacation destination | ○ | ○ | ○ | ○ | ○ |
| In planning a tour, I feel media is a very authentic source of information | ○ | ○ | ○ | ○ | ○ |
| Developments reported by media can change my opinion about a destination | ○ | ○ | ○ | ○ | ○ |

**13. What is your attitude towards the Chengdu government's initiatives? [Choose one for each row]**

|  | Strongly disagree | Disagree | Neutral | Agree | Strongly agree |
| --- | --- | --- | --- | --- | --- |
| Quality of infrastructure in Chengdu (public transport, roads, etc.) at the destination is satisfactory | ○ | ○ | ○ | ○ | ○ |
| I think the Chengdu government’s policies/regulations are favorable for tourists | ○ | ○ | ○ | ○ | ○ |
| I think the Chengdu government is committed to promoting the destination’s positive image | ○ | ○ | ○ | ○ | ○ |
| Services I received from Chengdu’s public servants (including tourism police, etc.) were satisfactory | ○ | ○ | ○ | ○ | ○ |

**14. What is your evaluation of Chengdu's tourism image? [Choose one for each row]**

|  | Strongly disagree | Disagree | Neutral | Agree | Strongly agree |
| --- | --- | --- | --- | --- | --- |
| I think Chengdu has a better image than other tourism destinations | ○ | ○ | ○ | ○ | ○ |
| I think the overall travel experience Chengdu provides is able to meet my needs | ○ | ○ | ○ | ○ | ○ |
| I would recommend Chengdu as a favorable destination | ○ | ○ | ○ | ○ | ○ |

**15. What is your intention to travel to Chengdu? [Choose one for each row]**

|  | Strongly disagree | Disagree | Neutral | Agree | Strongly agree |
| --- | --- | --- | --- | --- | --- |
| I am interested in going to Chengdu for travel | ○ | ○ | ○ | ○ | ○ |
| I will travel to Chengdu in the future | ○ | ○ | ○ | ○ | ○ |
| There is a high probability that I will travel to Chengdu in the future | ○ | ○ | ○ | ○ | ○ |
