# Supplementary material for: The mediation of perceived risk’s impact on destination image and travel intention: An empirical study of Chengdu, China during COVID-19
Source: PLoS One. 2022 Jan 7;17(1):e0261851. doi: 10.1371/journal.pone.0261851 (PMC8741017; doi:10.1371/journal.pone.0261851)
Supplement: Attachment — Submitted filename: Response to Reviewers.doc [file pone.0261851.s003.doc]

**Response to Reviewers**

Thank you for your valuable comments concerning our manuscript entitled “The Mediation of Perceived Risk’s Impact on Destination Image and Travel Intention: An Empirical Study of Chengdu, China during COVID-19”. These comments are all very helpful for the revision and improvement of our paper, as well as regards the guiding significance of our research. We have studied the comments carefully and made corrections which we hope will meet with approval. Our responses to these comments are as follows:

**Response to Reviewer 1**

**[General Comment]** I thoroughly enjoyed reading the manuscript and really appreciate the detailed literature review to build and support the hypotheses and excellent theoretical and analytical approaches and the discussion of the results. While I have no doubt about the merit of the research and its methodological rigor.

**Response:** Thank you very much.

**[Comment 1]** As a reader from outside PR China, I would have liked to know bit more about study site through location map.... Similarly the spread of respondents on a map would help to understand geographical location of respondents and distance from the study site.

**Response:** We apologize for the lack of clarity regarding geographical location in this manuscript and any inconvenience this caused in your reading. We have added a map of Chengdu that shows its districts, marking the location of key sites and medium-risk areas mentioned in this study, as well as the distribution of study respondents across China (P5, fig.1).

**Figure 1.** Study site

**[Comment 2]** General variation in local tourist numbers in different months before and after COVID.
Response: We appreciate your suggestion to include data on tourist visits before and during COVID-19. Indeed, this data would add depth to our study. During the writing process, and even to this day, we have spent a lot of time and effort to find the relevant month-by-month tourist visit numbers. Unfortunately, all publicly available data we have found is not precise to the month. We have contacted the Sichuan Provincial Department of Culture and Tourism and requested this data, but so far, have not received a reply. At the very least, we have annual tourist volume and growth rate data for Chengdu over the past ten years, and have mapped the trend of change (P6, Fig. 2), which will add at least some frame of reference to our study.

**Figure 2.** Annual tourist volume for Chengdu over the past ten years

**[Comment 3]** Similar some more description about the ‘Questionnaire Star’ and/or a weblink for the readers to know its recommendation process etc.

**Response:** Thanks for your valuable suggestion. In response to your concerns, we have added a description of the platform ‘Questionnaire Star’ and associated weblink: “Questionnaire Star” ([https: //www.wjx.cn/](https://www.wjx.cn/)) is a relatively well-established survey website dedicated to the self-design of questionnaires and related services in China. It is equipped with a wealth of questionnaire-style templates which editors can modify and operate independently. It provides complete output functionality, delivering survey results in various formats such as Word, PDF, and Excel. As of October 2021, the website has 2.6 million registered users. A total of 136 million questionnaires have been published on the questionnaire star platform, and 10.778 billion answer sheets have been collected. On average, more than 1 million people fill out questionnaires on the questionnaire star platform every day. Its paying customers cover more than 30,000 companies and 90% of universities in China” (P18-19, lines 356-378).

In addition, we have described Questionnaire Star’s recommendation process (which is automatic and random, automatically implemented by the platform’s system): “After applying the ‘Questionnaire Recommendation Service’ through Questionnaire Star, the online system randomly invited people from its 2.6-million-sample database to fill in their responses” (P19, lines380-382).

**[Comment 4]** Regarding demographic and related variables, I would like to mention that family composition and age of dependent kids or other family members could have provided useful information - though part of issue might have been addressed in social risks SOC2. The respondents have not been asked about frequency of their travel in before corona period as that could have explained how an otherwise frequent traveler responds. Similarly those have already visited the study site might have a different perception about intent to travel and destination image etc. Similarly it has not been captured that those form nearby areas who have been frequently visiting the have more or less urge than distant respondents and a variation in their responses.

**Response:** We are in full agreement with your comments on this matter.However, we designed our study to account for a limited number of variables—including the demographic variables of gender, age, educational level, marital status, industry, occupation, and source location, the travel behavior variable of whether one has participated in tourism activities since the outbreak of COVID-19, and travel experience (never traveled, traveled once, traveled multiple times). As the variables you put forward were not considered back when we designed the questionnaire, it is impossible to take them into account at this point in this study. While this is a shame, the questions your raise are also not essential to our main aim of improving understanding of Chengdu’s rebound in the complete prevention and control of COVID-19. Though we do acknowledge the deficiency, and take this as a warning and consideration for more thorough research in the future, it is impossible to go back collect this data for the research already done. Your concerns are of importance for our further study, and we will show the results in our next paper. We would be very grateful if you could continue to propose comments for our next article.

**[Comment 5]** The questionnaire is well elaborated, however some instrument items like in Social Risk factor (SOC1, SOC2, SOC3) or Equipment Risk (EQ1, EQ2, EQ3) seems confusing and there may be high likelihood that the respondent might have been confused in responding some items (For example....) I believe the questionnaire was in Chinese language and the translation in English could have created some confusion.

**Response:** We sincerely apologize for any misunderstanding caused by the language used or translation of terms. As you have indicated, our questionnaire was indeed conducted in Chinese. To avoid just such imprecision in language, we specially referred to similar research that was initially conducted in Chinese language and then published in English, for example the article “How to influence rural tourism intention by risk knowledge during COVID-19 containment in China: Mediating role of risk perception and attitude”, which was published in the International Journal of Environmental Research and Public Health (2020, Vol 17, Issue 10). Thus we aimed to remain consistent with existing conversations using these terms. As for the Chinese terms themselves, we did not receive any confusing feedback from interviewees during the pre-investigation and formal testing phases that would suggest a misunderstanding on their part.

Thank you again for your time and very thoughtful comments.

**[General Comment]** Despite these minor comments, the study is an excellent effort to capture COVID-19 related barriers to tourism through mediated and direct impacts.

**Response:** Thank you very much.

**Response to Reviewer 2**

**[Comment 1]** Abstract should be revised and add more results and practical policy implications.

**Response:** We greatly appreciate your suggestion. We have added more results and practical policy implications in a revised abstract. “This study is significant because it provides a theoretical interpretation of tourism risk, elucidates the mechanisms or paths by which perceived risk affects travel intention, and expands a framework for research on destination image and travel intention into the realms of psychology, political, and communication science. It additionally encourages people to pay greater attention to the negative impact of crises and focuses on the important role of internal and external responses in crisis management, which can help improve the effectiveness of crisis management and promote the sustainable development of the tourism industry.” (P1-2, lines 27-34).

**[Comment 2]** Try to reduce some information in the first section, it is very lengthy.

Response: We have adjusted the format of the article in consideration of the journal’s requirements and have simplified the presentation, making the language more concise. By asking questions and offering a narrative, we have made the article more interesting and attractive of readers’ attention.

**[Comment 3-5]** In methodology, what is reliability of data?How biasness was addressed and set?why this sample number was selected and how? which method used and why?

**Response:** We firmly concur with your suggestion. We think that these are different aspects of the same issue, so we address them here together.

Because there is no clear or uniform requirement for sample size in statistics, the common standard is 5-10 times the scale items. As our questionnaire contains a total of 34 scale items in our questionnaire, a suitable sample size would be between 170 and 340. Some scholars have also constructed a formula for calculating the total sample size. Wu believes that when the sample population is quite large or infinite, the sample size formula is as below (1)

$n\geq\left(\frac{k}{\alpha}\right)^{2}p\left(1-p\right)$ (1)

where *n* represents sample size. Usually *p* and αare set respectively to 0.5 and 0.05, because the most reliable sample size can be obtained when 0.5 and 0.05 is set. In this study, α was at 95% confidence level, that is, *p*=0.5. And the confidence level used in interval estimation is 1-0.05=0.95. The time quantile is *k*=1.96. Therefore, the formula yielded an n of 384.16 as the minimum acceptable sample size. Using convenience sampling, a total of 413 samples were obtained in this study, thus meeting the above-described sample number requirements for surveys (P18-19, lines 368-378).

Second, in terms of sample distribution, the respondents come from 91 cities in 32 of China’s provinces or regions. Most tourists are from Sichuan Province. Also, the respondents were employed in 15 occupations across 24 industries. For these reasons, we believe that the sampling is representative and the research has excellent external validity. We have added a distribution map of respondents across China (Fig.1).

In the process of data analysis, we conducted reliability and validity analysis. The results showed that the internal reliability of the data to be excellent (P21-23, lines 430-450).

**[Comment 6 and 8]** Discussion heading and after that “findings”, inappropriate. Discussion should not contain findings. only discuss and compare with other such similar studies.Add table on the comparison of such studies in Sichuan or nearby provinces in China and if not then from other cities of China.

**Response:** Thank you for making these important points. We have adjusted the corresponding format and content in accordance with the journal format of PLOS One. Indeed, it would be more convincing if we were able to compare against similar studies done in Sichuan or nearby provinces or other cities in China. However, when checking the articles included in SCI, SSCI and CSSCI, we found that there are very few such studies. At present, most research focuses on the whole of China or more narrowly on a certain type of tourism (for example, rural tourism). Few articles take a specific city as their example. In particular, destination research on the post-COVID rebound has yet to be published. Therefore, we seek for the editor’s tolerance and understanding. Many thanks for your kind help! We do our best to organize similar studies in the following table.

Table 6 Similar studies in China

| Researcher(s) | Study site | Findings and relevant conclusions |
| --- | --- | --- |
| Zhu (2020) | China | Potential participants paid most attention to the performance realization and time cost of scenic spots, while the psycho-social risk posed by COVID-19 had little impact. With regard to travel intention and recommendation intention, the negative impacts of risk aversion attitude were more considerable compared to risk perception. Meanwhile, the parallel mediating effect of risk perception and risk aversion attitude in rural tourism needed to be taken into consideration together. |
| Ahmed (2020) | China | Higher rate of anxiety, depression, hazardous and harmful alcohol use, and lower mental wellbeing than usual ratio. |
| Luo (2020) | Hong Kong | The fear of COVID-19, travel anxiety and risk attitude negatively impact travel intention. Furthermore, travel anxiety and risk attitude moderate the indirect impacts between fear of COVID-19 and travel intention. |
| Wang (2020) | Jilin City (Jilin Province) | SARIMA model is established to analyze the impact of COVID-19 on China’s tourism revenue, and it is concluded that China should focus on improving the quality and upgrading of domestic tourism in the future. The Challenges and Countermeasures for Tourism Industry of China were discussed. |
| Wang (2020) | Henan Province | The impact and formation mechanism of the Novel Coronavirus Pneumonia( NCP) on the tourism industry in Henan Province were investigated. In the period of “post-NCP “,tourism development will return to the nature of health, people travel slowly and hot, tourism recovery will be a difficult recovery process. |
| Yang (2020) | Hubei Province | During the different stages of COVID-19, including discovery, outbreak, and subsidence, the overall impact of urbanization on the economy in Hubei Province was first positive, then became negative, and finally gradually increased. This process had significant industrial and urban heterogeneity, which was mainly manifested in losses in tourism and catering industries that were significantly greater than those in the audio-visual entertainment and digital office industries. |
| Qiao (2021) | China | Mass media had a positive influence on South Korean residents’ perception of China’s image, a negative influence on residents’ concerns, and a positive influence on residents’ behavioral intentions for travel overseas. |
| Wong (2021) | Macao | A survey instrument was designed to evaluate resident satisfaction with six enforcement actions taken by the Macao government during the COVID-19 pandemic. Each factor had a different effect on satisfaction and trust that influenced the resident support for tourism recovery. |
| Lu (2021) | Sichuan Province | The hospitality industry sector, the most severely affected sector in five industries, by cash flow pressure. |
| Sun (2021) | Hainan Province, Hubei Province, Shanghai, Beijing | Four game models for epidemic prevention and control and tourism recovery are proposed, combined with the Logistic growth curve, to predict the recovery of passenger traffic in different cases, and use it as tourism popularity to deduce tourism recovery. The recovery of passenger traffic is subject to the epidemic prevention and control policy, but the policy. The intensity of the impact on passenger traffic is less than that of the epidemic. |
| Yang (2021) | Hengyang (Hunan Province) | Travel time perception, tourism function perception, tourism satisfaction, tourism risk perception and tourism willingness will affect tourism well-being. |

**[Comment 7]** For conclusions also follow my comments as for abstract.

**Response:** We are appreciative of your insightful suggestion. We have added more results and practical policy implications in the conclusions section (P34-36, lines 597-647). Thank you again. It was your valuable and insightful comments that made improvements in the current version possible.

**[Comment 9-10]** Also better to give a map of Chengdu and show there data collection points and sampling. Add more figures if possible for better understanding and presentation.

**Response:** We sincerely approve of your suggestion, and have added the following map of Chengdu. The map shows data collection points as well as sampling distribution. Inspired by your comments, we specially mark the six medium-risk areas (during the relevant timeframe) in Chengdu. We have also found Chengdu’s annual tourist volume and tourism growth rate data for the past ten years, and show the trend of change in a graph below (Fig. 1, Fig.2). Thank you for your valuable suggestions. These comments are all very helpful for revising and improving our paper, as well as providing important guiding significance to our research.

Figure 1. Study site

Figure 2. Annual tourist volume for Chengdu over the past ten years
